# Retraction: LINC00636 promotes lymph node metastasis and of cervical cancer through target NM23

**DOI:** 10.1042/BSR-2020-0367_RET

**Published:** 2024-01-31

**Keywords:** cervical cancer, LINC00636, metastasis, NM23

This article is being retracted from *Bioscience Reports* at the request of the Editor-in-Chief and the Editorial Board following exchanges with two of the four listed authors, Qiang Lu and Yan Luo. Qiang Lu and Yan Luo both contacted the journal via email addresses that differed to the one included with the submission, both stating that they should not be listed as authors of the article, that they were not involved with the research and that they had not consented to being authors on the article. Upon review of the case, the Editorial Office believe that the peer review process was also likely compromised for this article.

All authors listed on the article (using the email addresses provided at the time of the articles submission) have been contacted by the Editorial Office to respond to the authorship dispute but have not responded. Given the extent of the authorship issues raised, the Editorial Board stand by the decision to retract the article.

